# Influence of Different ECM-Like Hydrogels on Neurite Outgrowth Induced by Adipose Tissue-Derived Stem Cells

**DOI:** 10.1155/2017/6319129

**Published:** 2017-12-03

**Authors:** E. Oliveira, R. C. Assunção-Silva, O. Ziv-Polat, E. D. Gomes, F. G. Teixeira, N. A. Silva, A. Shahar, A. J. Salgado

**Affiliations:** ^1^Life and Health Sciences Research Institute (ICVS), School of Medicine, University of Minho, Campus de Gualtar, 4710-057 Braga, Portugal; ^2^ICVS/3B's-PT Government Associate Laboratory, Braga, Guimarães, Portugal; ^3^NVR Research Ltd., Ness-Ziona, Israel

## Abstract

Mesenchymal stem cells (MSCs) have been proposed for spinal cord injury (SCI) applications due to their capacity to secrete growth factors and vesicles—secretome—that impacts important phenomena in SCI regeneration. To improve MSC survival into SCI sites, hydrogels have been used as transplantation vehicles. Herein, we hypothesized if different hydrogels could interact differently with adipose tissue-derived MSCs (ASCs). The efficacy of three natural hydrogels, gellan gum (functionalized with a fibronectin peptide), collagen, and a hydrogel rich in laminin epitopes (NVR-gel) in promoting neuritogenesis (alone and cocultured with ASCs), was evaluated in the present study. Their impact on ASC survival, metabolic activity, and gene expression was also evaluated. Our results indicated that all hydrogels supported ASC survival and viability, being this more evident for the functionalized GG hydrogels. Moreover, the presence of different ECM-derived biological cues within the hydrogels appears to differently affect the mRNA levels of growth factors involved in neuronal survival, differentiation, and axonal outgrowth. All the hydrogel-based systems supported axonal growth mediated by ASCs, but this effect was more robust in functionalized GG. The data herein presented highlights the importance of biological cues within hydrogel-based biomaterials as possible modulators of ASC secretome and its effects for SCI applications.

## 1. Introduction

The current care of SCI patients is mostly palliative. Thus, it is extremely important to develop new strategies that support both structural and functional restoration of the damaged or lost tissue. For this purpose, tissue engineering approaches, using biomaterials, stem cells, and often neurotrophic factors, are being extensively explored [1]. Biomaterials, particularly hydrogels, are interesting for SCI approaches mainly due to their physical properties that resemble central nervous system (CNS) soft tissues. Moreover, their porous structure allows molecule diffusion and the possibility to establish a three-dimensional (3D) environment that mimics the living tissues. These biomaterials are widely used to support cell transplantation [2]. For this purpose, natural hydrogels are an interesting tool, as they are mostly constituted by extracellular matrix (ECM) molecules or can be easily functionalized with them [3]. Among the variety of available natural hydrogels, this study will focus on the use of gellan gum, NVR-gel, and collagen as matrices for cell encapsulation. Gellan gum (GG), a linear anionic polysaccharide with a free carboxylic group per repeating units, was already reported to be biocompatible and nontoxic after injection in a hemisection SCI rat model [4]. Moreover, it was also shown to be suitable for functionalization, as its modification with a fibronectin-derived peptide (GRGDS) promoted higher cell adhesion and viability in comparison to unmodified GG [5]. Regarding NVR-gel, this is a biocompatible and biodegradable scaffold composed of hyaluronic acid (HA) and laminin [6]. While HA has a vital role in cell morphogenesis and proliferation [7], laminin provides an adhesive and growth-promoting substrate [6]. In fact, this gel was shown to be an excellent 3D milieu for the growth of neuronal tissue in culture [6]. Finally, collagen has been reported as the main constituent of almost all ECMs. Together with its biocompatibility, collagen enables to form adhesive and permissive substrates for cell survival and neurite outgrowth [8]. This is supported by studies where, for example, neural stem/progenitor cells (NPCs) cultured on type I collagen hydrogel were found to develop functional synapses and form neuronal networks [9]. On the other hand, the use of stem cell-based therapies in SCI regenerative strategies has also shown interesting results. Within the different cell populations available, MSCs have been put forward as a possible choice [10, 11]. The most relevant feature of these cells is the active secretion of bioactive molecules (e.g., growth factors, cytokines, and exosomes), nowadays known as secretome. Indeed, studies have already demonstrated that after transplantation of MSCs (from different sources) on SCI models, neurotrophic factors such as brain-derived neurotrophic factor (BDNF), vascular endothelial factor (VEGF), interleukin-6 (IL-6), neurotrophin 3 (NT-3), basic fibroblast growth factor (bGFG), stem cell factor (SCF), hepatocyte growth factor (HGF), and glial cell line-derived neurotrophic factor (GDNF) were secreted, which was correlated with prominent increase of axonal regeneration and functional outcomes [3, 12, 13]. Despite these encouraging results, MSC transplantation still faces the problem of poor cell engraftment and survival in the lesion site [3]. Under this context, ECM-like hydrogels such as GG, NVR, and collagen could play a crucial role in overcoming these limitations. Indeed, by using these systems, it would be possible to extend cell survival within the injured site, as well as to modulate their behavior through the cues presented by the different cell adhesion peptides to MSCs. Having this in mind, in the present work, we aimed to evaluate the efficacy of the three referred natural hydrogels, namely, GG (functionalized with a fibronectin peptide), collagen, and NVR-gel in supporting the viability, metabolic activity, and gene expression of MSCs isolated from the adipose tissue 1 (adipose tissue-derived stem cells (ASCs)). Moreover, the effects of these hydrogels as modulators of ASC impact on axonal growth will be also addressed using dorsal root ganglion (DRG) explant cultures.

## 2. Materials and Methods

### 2.1. Adipose-Derived Stem Cell (ASC) Culture

Human ASCs (female, 25 years old, BMI = 27.8) were kindly provided by Professor Jeffrey Gimble (Tulane University Center for Stem Cell Research and Regenerative Medicine and LabCell LLC, New Orleans, Louisiana, USA) and cultured in *α*-MEM (Invitrogen, USA) medium [supplemented with sodium bicarbonate (NaHCO^3^, Merck, USA), 10% of fetal bovine serum (FBS) (Biochrom AG, Germany), and 1% of penicillin-streptomycin antibiotic (Invitrogen, USA)] at 37°C and 5% CO^2^. ASCs used and disclosed the following marker phenotypes: CD29: 93.63%; CD105: 97.09%; CD44: 87.50%; CD73: 92.86%; CD90: 88.99%; CD45: 2.11%; and CD34: 7.60%.

### 2.2. Hydrogel Preparation

#### 2.2.1. Gellan Gum

Gellan gum (GG, Sigma, USA) was modified with the fibronectin-derived peptide (GRGDS) as previously described [3]. First, GG was dissolved in 2-(N-morpholino)ethanesulfonic acid (MES) buffer (100 mM, pH 5.5, Sigma, USA) and stirred for 48 h at 37°C. 4-(4,6-Dimethoxy-1,3,5-triazin-2-yl)-4-methylmorpholinium chloride (DMT-MM, Sigma, USA) and furfurylamine (Acros Organics, Belgium) were then added in a 4 : 1 M ratio (of each reagent relative to the eCOOH groups in gellan gum) and stirred at 37°C for 48 h. The solution was then dialyzed (Mw cutoff 12–14 kDa, Spectrum Labs, USA) alternately with distilled water and PBS (0.1 M, pH 7.2) for 5 days. After lyophilization, furan-modified GG (furan-GG) was obtained as a white powder.

The immobilization of GRGDS peptide (AnsSpect, USA), previously modified with a maleimide group (mal-GRGDS), into the furan-GG was performed by Diels-Alder chemistry, originating the GG-GRGDS. For that, furan-GG was dissolved in MES buffer (100 mM, pH 5.5) at 37°C (1.2 mg/ml). The mal-GRGDS was then added to furan-GG in a 5 : 1 maleimide : furan molar ratio and stirred at 37°C for 48 h. The mixture was dialyzed (Mw cutoff 12–14 kDa) for 5 days as described above. The water was then removed by lyophilization and GG-GRGDS was obtained as a white powder.

#### 2.2.2. NVR-Gel

NVR-gel (NVR Labs, Israel) is mainly composed of two components: hyaluronic acid (HA, 3 × 10^3^ KDa, BTG Polymers, Israel) and laminin (Sigma, USA). To prepare this hydrogel, HA 1% (*w*/*v*) and laminin were diluted in cell culture medium, at a concentration of 0.3% (*v*/*v*). The solution was then thoroughly mixed to obtain a liquid viscous hydrogel.

#### 2.2.3. Collagen

Collagen gels were prepared as described by Allodi et al. [14]. Briefly, gels were prepared by mixing 450 *μ*l of collagen type I (BD Biosciences, USA), 50 *μ*l of Dulbecco's modified Eagle medium 10× (DMEM 10×, Sigma, USA), and 2 *μ*l of 7.5% NaHCO^3^ solution. Then, hydrogels were kept in the incubator for 2 h at 37°C and 5% CO^2^ to allow the gelification process to occur.

### 2.3. Hydrogel Preparation and ASC Encapsulation

#### 2.3.1. Gellan Gum

Prior to ASC encapsulation, the lyophilized GG-GRGDS was sterilized by UV light for 15 min. Then, GG-GRGDS was dissolved in ultrapure water at 1% (*w*/*v*) concentration and placed to stir at 40°C overnight. Next day, GG was dissolved in ultrapure water at 1% *w*/*v* concentration, placed to stir at 90°C for 30 min, and then sterilized by filtration. GG-GRGDS and GG were then mixed together in a 50 : 50 proportion. Just before cell encapsulation, calcium chloride [CaCl^2^; 0.3% (*w*/*v*)] was added to the GG-GRGDS/GG mixture in a proportion of 1 : 10, to promote the ionic crosslinking of the hydrogel.

ASCs were obtained as previously described. Cells were encapsulated within the hydrogel by gently mixing the cell pellet with the desired volume of gel, at a concentration of 6 × 10^5^ cells/ml until a homogeneous solution was obtained. The hydrogel containing the cells was placed on cell culture wells in drops of 50 *μ*l per chamber/well and cultured from 4 to 7 days.

#### 2.3.2. NVR-Gel

After mixing the hydrogel with cell culture medium, a liquid viscous gel was obtained. The desired volume of hydrogel was added to the cells' pellet at a concentration of 6 × 10^5^ cells/ml and mixed to uniformly distribute the cells. Then, 250 *μ*l of the gel containing the ASCs was added to cell culture wells and kept in culture for 4 and 7 days.

#### 2.3.3. Collagen

After collagen hydrogel gelification, the cell pellet was resuspended with the desired volume of hydrogel at a concentration of 6 × 10^5^ cells/ml. The hydrogel containing the cells was placed on cell culture wells in 50 *μ*l drops and placed in the incubator for 1.5 h at 37°C and 5% CO2 for the gelification process to occur. Cell culture medium was then added to the wells and kept in culture for 4 and 7 days.

### 2.4. MTS

The MTS assay relies on the reduction of the tetrazolium compound 3-(4,5-dimethylthiazol-2-yl)-5-(3-carboxymethoxyphenyl)-2-(4-sulfophenyl)-2H-tetrazolium (MTS, Promega, USA) to formazan by viable cells. After 7 days of culture, the cell culture medium was replaced by serum-free medium (DMEM, Invitrogen, USA), containing MTS in a 5 : 1 ratio and incubated for 3 hours at 37°C and 5% CO^2^. For sample analysis, triplicates of each sample were placed in wells of a 96-well plate, allowing the optic density to be measured by spectrometry (at 490 nm) in a microplate reader.

### 2.5. Quantitative Real-Time PCR

After ASC encapsulation and culture on different hydrogels, a RT-PCR was performed to analyze the expression levels of specific genes involved in axonal growth, namely, BDNF, VEGF, NGF, and GDNF.

#### 2.5.1. RNA Extraction

After 7 days of cell culture, the total RNA from ASCs encapsulated in the hydrogels was extracted. For that, 6 gels were combined to obtain a more reliable sample. Initially, 1 ml of Trizol solution (Life Technologies, USA) was added to the hydrogels containing the cells, and after a mechanical dissociation of the gels, a cell pellet was obtained. The samples were then collected in Eppendorf tubes. The cells' pellet was then incubated with chloroform (Sigma, USA) for 3 min at room temperature (RT) and centrifuged at 8000 rpm for 15 min, at 4°C. After this, the presence of three layers in the Eppendorf tube was possible to observe: an aqueous phase on top, an interphase, and an organic phase in the bottom. The aqueous phase, containing the RNA, was transferred into new Eppendorf tubes and precipitated by adding isopropanol (Carlos Erba Reagents, France). The samples were then left to rest for 10 min at RT. Afterwards, the samples were centrifuged (9000 rpm, 15 min, 4°C), the RNA pellets were washed with 75% ethanol and centrifuged again (5000 rpm, 5 min, 4°C). Then, the pellets were dissolved in nuclease-free water and the RNA quantification was performed using Nanodrop ND-1000 spectrophotometer (Alfagene, Portugal), to adjust the sample concentration to 500 ng/*μ*l.

#### 2.5.2. cDNA Transformation and Quantitative Real-Time PCR

The synthesis of the cDNA was performed using iScriptTM cDNA Synthesis Kit (BioRad, USA). First, 1 *μ*g of purified RNA was used as a template and the volumes were normalized with nuclease-free water. A reaction mix containing the RNA sample, 5× iScript reaction mix, and iScript reverse transcriptase was prepared and added to the samples. Periods of sample incubation of 5 min at 25°C followed by 30 min at 42°C and 5 min at 85°C in a thermal cycler (Applied Biosystems, USA) were performed. To proceed with the quantitative gene expression analysis, the cDNA was subject to PCR amplification using Eva Green technology (Ssofast Evagreen Supermix, BioRad, USA) on the CFX96 Real-Time system (BioRad, USA).

Finally, a reaction solution was prepared by mixing 5 *μ*l of Ssofast Evagreen Supermix, 10 *μ*M of the primer respective to each tested gene, in forward and reverse, and 1 *μ*l of cDNA. After adding the reaction solution to the template, specific cycling conditions were applied to samples, such as 30 s at 95°C of enzyme activation, followed by 40 cycles of 5 s at 95°C for denaturation, 5 s at 60°C for annealing, and 5 s at 72°C for extension step. The HMBS gene (XM_017017629.1) was the house-keeping gene herein used, and the tested genes were the BDNF (NG_011794.1), VEGF (AY500353.1), NGF (NG_007944.1), and GDNF (NG_011675.2), as described in Table 1.

### 2.6. DRG Coculture with ASCs in 3D Hydrogel-Based Environment

As previously described, ASCs were encapsulated within the hydrogels at a concentration of 6 × 10^5^ cells/ml. Four replicates of 50 *μ*l of each hydrogel were maintained in complete *α*-MEM for 24 hours, to let cells grow within the gel. After this time, DRGs from P5 Wistar-Han rat pups were dissected as previously described [15] and placed on top of the ASC-containing hydrogels. Cultures were kept for 7 days in neuron medium (Invitrogen, USA; neurobasal medium supplemented with B27, L-glutamine, glucose, and pen-strep). DRG culture on top of hydrogels without cells was used as control.

### 2.7. Immunocytochemistry and Phalloidin/DAPI Staining

After 4 and 7 days of cell culture, ASC and DRG cultures were prepared for phalloidin/DAPI staining and immunocytochemistry (ICC), respectively.

For ASC staining, phalloidin (Sigma, USA) was used to stain the F-actin filaments within the cells' cytoskeleton, while DAPI (Invitrogen, USA) was used to mark the cells' nucleus. Samples were fixed with 4% (*v*/*v*) paraformaldehyde (PFA) for 45 min at RT. After washing with PBS 1×, phalloidin (0.1 *μ*g/ml; Sigma), associated with Alexa-Fluor 594, and DAPI (1 *μ*g/ml; Invitrogen) were added to cell for 45 min at RT.

For ICC, an antibody for neurofilament (NF) was used to stain the intermediate filaments of DRG neurons. For that, the following antibodies were used: the mouse monoclonal anti-rat neurofilament 200 kDa (Millipore, USA) as the primary antibody and the Alexa fluor 488 goat anti-mouse IgG (H + L) (Invitrogen, USA) as the secondary antibody. Samples were fixed with 4% (*v*/*v*) PFA for 45 min at RT. For cell membrane permeabilization, samples were incubated with 0.3% Triton X-100 (Sigma, USA) for 10 min at RT and washed 3 times with PBS 1×. Samples were then incubated with a blocking buffer solution [PBS 1× containing 10% FBS] for 90 min at RT. Finally, samples were incubated with the primary antibody for 48 h at 4°C. After washing three times with PBS 1×, samples were incubated with the secondary antibody overnight at 4°C.

### 2.8. Quantification of Cell Densities and Axonal Growth

For cell density measurement and morphology analysis, ASC-containing hydrogels were imaged by confocal point-scanning microscopy (Olympus FV1000). The number of cells present in the hydrogels was inferred by counting the number of DAPI^+^ cells in six regions taken at random in each gel sample, using the cell counter plugin of the ImageJ software.

For DRG axonal growth quantification, DRG cultures on top of the hydrogels were imaged by fluorescence microscopy (Olympus BX-61 Fluorescence Microscope, Olympus, Germany). Axonal growth was inferred by quantifying the area occupied by the neurites within the hydrogels. For that, three representative micrographs of each sample were taken and later analyzed using ImageJ software. The image scale was first set and converted to 8 bit and binary, which will convert the green fluorescence (neurofilament staining) into a black image with a white background. The body of the DRG was then excluded. Thereafter, through the “analyze particles” program's setting, the area occupied by the neurites was automatically calculated considering the image dark background as contrast.

### 2.9. Statistical Analysis

All statistical analyses were performed using GraphPad Prism (version 5.0; GraphPad Software, USA). Differences among groups were assessed using *t*-test or one way ANOVA test, followed by a Turkey post hoc. A *p* value of ≤0.05 (95% confidence level) was set as the criteria for statistical significance (^∗^).

## 3. Results

### 3.1. ASC Survival and Metabolic Activity in the Three Matrices

In order to assess whether the GG-GRGDS, NVR-gel, and collagen have the capacity to promote ASC survival, viability, and proliferation, cells were encapsulated on the three hydrogels at a cell density of 6 × 10^5^ cells/ml and cultured for 7 days in serum-containing *α*-MEM. 2D cultures were used as control. At the end of cell culture, phalloidin/DAPI staining was performed.

**Figure 1:**
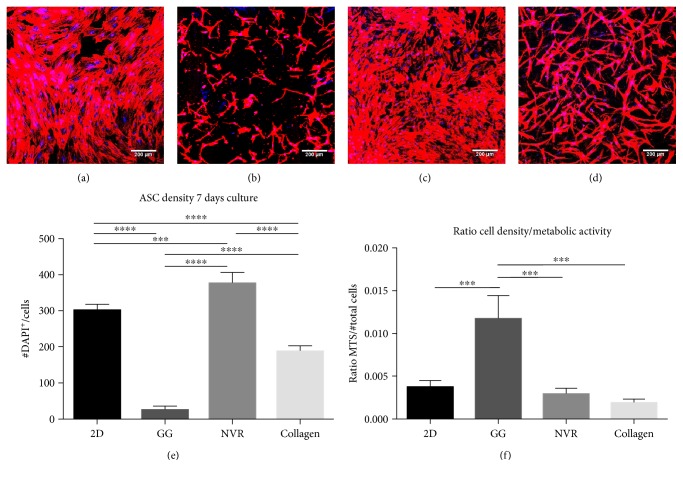
ASC encapsulation within the three matrices. ASC morphology was analyzed in (a) 2D cultures, (b) GG-GRGDS, (c) NVR-gel, and (d) collagen. Moreover, (e) cell density and (f) the ratio cell density/metabolic activity were also assessed in serum-containing *α*-MEM. Immunostaining for phalloidin (red) and DAPI (blue). Mean ± SD; *n* = 4 per condition; *p* < 0.05. Scale bar: 200 *μ*m.

All hydrogels were found to support ASC survival and proliferation, as observed in Figures 1(a), 1(b), 1(c), and 1(d). ASCs disclose their typical spindle-like shape morphology. Nevertheless, cell behavior was different within each matrix, as the number of cells (DAPI^+^ cells) in the three hydrogels and control was significantly different (Figure 1(e)).

In addition, a MTS assay was performed to assess the metabolic activity of ASCs in the three hydrogels. Results were presented as cell density/metabolic activity ratio (Figure 1(f)). The cells within the GG-GRGDS gels had a significantly higher metabolic activity when compared to 2D cultures, and NVR-gel, and collagen.

### 3.2. Pilot Screening on the Effects of GG-GRGDS, NVR-Gel, and Collagen on the Gene Expression of Neurotrophic Factors

ASC gene expression on some neurotrophic factors relevant for axonal growth—BDNF, VEGF, NGF, and GDNF—was analyzed using quantitative real-time PCR in cells that were encapsulated on the different hydrogels.

**Figure 2:**
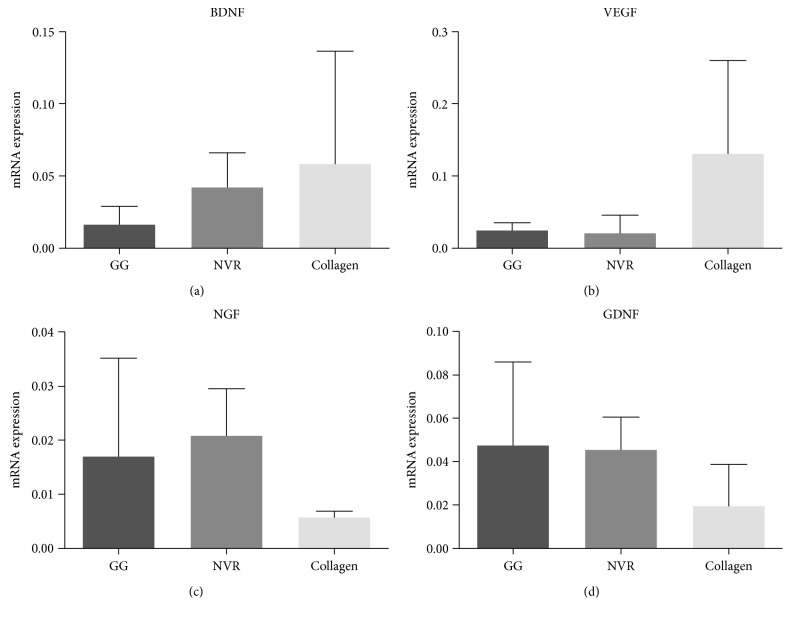
mRNA levels of BDNF, VEGF, NGF, and GDNF expression in ASCs cultured in GG-GRGDS, NVR-gel, and collagen after 7 days of cell culture. HMBS was the house-keeping gene herein used. Mean ± SD; *n* = 3.

No statistical differences were observed among gene expression of ASCs in the different hydrogels for all the tested growth factors, as denoted in Figure 2. Nevertheless, BDNF and VEGF (Figures 1(a) and 1(b), resp.) appear to have a higher expression in ASCs cultured in collagen gels, which contrasts with lower expression profiles in GG-GRGDS and NVR-gel. In turn, NGF and GDNF mRNA levels (Figures 1(c) and 1(d), resp.) tends to be higher and very similar in these hydrogels, when compared to collagen.

### 3.3. Impact of ASCs on Axonal Growth: Role of ECM-Like Hydrogels

**Figure 3:**
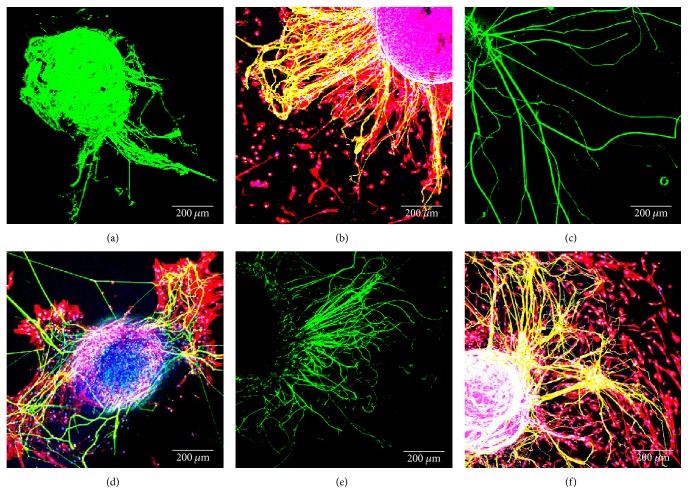
Coculture of ASC and DRG explants in serum-free conditions on GRGDS-GG (b), NVR-gel (d), and collagen (f). DRG monoculture on GG (a), NVR-gel (c), and collagen (e) was used as control. Immunostaining for neurofilament (green), phalloidin (red), and DAPI (blue). Scale bar: 200 *μ*m.

**Figure 4:**
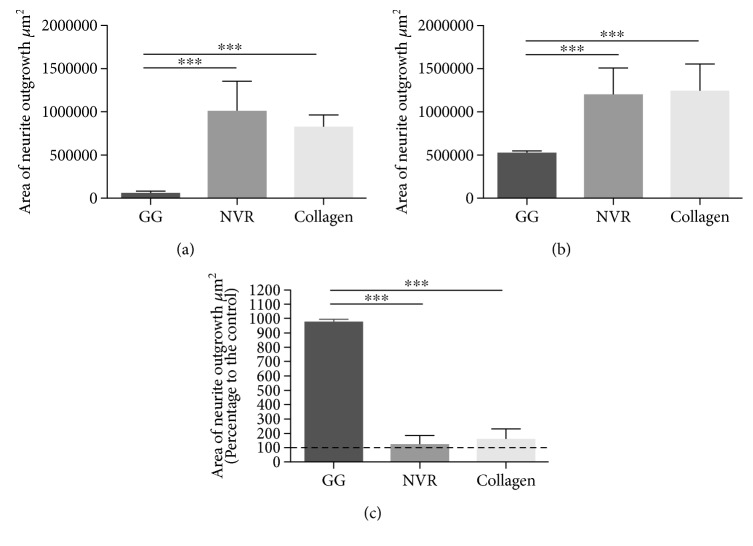
Quantification of the neurite outgrowth promoted by (a) hydrogels alone, (b) hydrogels plus ASCs, and (c) hydrogels plus ASCs regarding the respective controls. The mean area occupied by neurites (*μ*m^2^) was calculated using Neurite J plugin for ImageJ (NIH) software. Results presented as mean ± SD; *n* = 4 per condition.

A DRG *in vitro* model of neurite/axonal growth was used to evaluate the impact of ASCs encapsulated on the different gels. For this purpose, ASCs were firstly encapsulated on the different gels, and DRGs were placed on top of it. After 7 days of culture in neuron medium, neurofilament staining was performed and DRG axonal growth was quantified as previously described. The above-referred conditions are represented in Figures 3 and 4.

Upon quantification, DRG axonal growth was found to be promoted either by gel alone (GG: 53925.1 ± 33506.3; NVR: 996721 ± 706734; collagen: 813013 ± 301688) (Figures 4(a), 4(b), and 4(c)) and by gels plus cells (GG: 524567 ± 14206.1; NVR: 1.19 × 10^6^ ± 631584; collagen: 1.23 × 10^6^ ± 648435) (Figures 4(a), 4(b), and 4(c)). Even though GRGDS by itself was not able to promote high levels of neurite outgrowth, the addition of ASCs had a significant impact on its effect. Upon normalization against the respective controls, denoted in Figure 4(c), ASC-containing GG-GRGDS hydrogels were found to promote a robust and significant improvement of axonal growth (972.77 ± 26.34), while NVR and collagen shifts were marginal (119.72 ± 63.36; 151.52 ± 79.76, resp.).

These results suggest GG-GRGDS to be the most favorable for ASCs in the promotion of axonal growth by these cells.

## 4. Discussion

As previously stated, MSC transplantation is a promising therapy to tackle the complex and diversified events that occur in SCI, mainly due to their immunomodulatory capacity with prosurvival character and regenerative potential proven to be related with their secretome [11, 13, 16, 17]. However, the engraftment rate of transplanted cells in the lesion site is lower than the required to induce a regenerative response [18]. For that reason, hydrogels have been proposed as a substrate that promotes cell survival and proliferation and allows a constant secretion of molecules from MSCs in a local manner, thus dodging the need of repeated administrations [1].

In this study, we intended to test three different matrices—GG-GRGDS, NVR-gel, and collagen—as vehicles for ASCs. After encapsulating the cells in these hydrogels, all of them were found to support ASC survival, viability, and proliferation. As shown in Figures 1(a), 1(b), 1(c), and 1(d), ASCs were able to adhere to the 3D matrices, extending their processes and presenting their typical spindle-like shape morphology. Nevertheless, cells behave differently within each matrix, as can be inferred by differences observed in cell density after 7 days in culture (Figure 1(e)). In fact, the NVR-gel presented a statistically significant higher cell density when compared to 2D control and GG-GRGDS. Collagen follows NVR-gel, and the lowest cell density was observed for GG-GRGDS, all presenting statistical differences between them. The discrepancies observed in these results highlight the role of the ECM on cell adhesion, survival, and proliferation, besides demonstrating cell-type affinity to different ECM molecules. For example, the NVR-gel ability to promote higher cell proliferation is likely to be caused by the presence of the adhesion molecule laminin. In addition, this can also be triggered by the HA present in hydrogel's composition, which has high affinity to the cell surface receptor CD44 [19], documented to be present in MSCs [20]. In fact, Zhu et al. [21] evaluated the CD44-HA interaction in rat MSC migration and discovered that upon PDGF stimulation, CD44 expression in MSC was increased. Moreover, cell adhesion and migration was indeed proved to be dependent on that interaction.

As one of the main constituents of ECM, collagen is crucial for cell-cell and cell-ECM signaling and adhesion. Importantly, cell recognition of these ECM molecules is crucial for them to attach, survive, and proliferate. Therefore, this could explain why cells had easily proliferated in the collagen-based hydrogels. This is in accordance with a report that screened several proteins to uncover which molecules allowed a preferential binding of MSCs and the ones that avoided a strong adherence of fibroblasts. The results showed that MSCs bind with a higher efficiency to collagens (I, III, and IV) and laminin-111, when compared to fibroblasts. Moreover, the same study showed that both cell types bind with equally high efficiency to fibronectin. With this in mind, one can conclude that the presence of fibronectin peptides on the GG may justify the adherence, survival, and typical morphology of the ASCs on this matrix. This was indeed observed in a study performed by Silva et al. [3], where encapsulated BM-MSCs in a GRGDS-modified GG revealed higher cell proliferation and metabolic activity than unmodified gels. Moreover, cell secretome was also positively influenced by the presence of the peptide, as BM-MSCs seeded in GG-GRGDS induced a higher metabolic viability and neuronal density of primary cultures of hippocampal neurons than those seeded in the regular GG. The reason why this hydrogel presented the lowest response of ASCs from the three tested hydrogels might be owed to the peptide concentration on the hydrogel, which may perhaps be lower than the necessary to attain the desired effect. In fact, as described by Silva et al. [3], the engraftment success for this molecule into the backbone of GG is around 30–35%, meaning that the GG-GRGD hydrogel had decreased the levels of cell adhesion motifs when compared to collagen and NVR hydrogels.

Along with cell density measurements, the metabolic activity of ASCs in the three hydrogels was also observed to differ (Figure 1(f)). Interestingly, although GG-GRGDS presented the lowest cell density, the surviving cells had a significantly higher metabolic activity when compared with the remaining hydrogels and control. This is similar to the results obtained in the abovementioned study of Silva et al., as the presence of this peptide allowed BM-MSCs to survive within this modified GG hydrogel [3]. Regarding NVR-gel and collagen, ASC metabolic activity was very similar to control, suggesting no adverse effects of these hydrogels on ASCs. Nevertheless, the results obtained with the GG-GRGDS are of note. Altogether, these results indicate that the presence of different ECM molecules may exert varying responses on cellular functions by the same cell population. Moreover, they also show how ECM incorporation on scaffolds provides a higher cell response, which is advantageous for regenerative strategies using cell- and biomaterial-based therapies.

The observation that different hydrogels/ECM molecules influenced differently ASCs' viability and metabolic activity leads to question whether their gene expression on some neurotrophic factors relevant for axonal growth could also be different. For that reason, ASC mRNA levels of BDNF, VEGF, NGF, and GDNF were measured. BDNF, GDNF, and NGF belong to the neurotrophin family, which has an important role in neuronal survival and/or protection and axonal growth [22]. Some studies report BDNF to support motor and sensory neuron survival (neuroprotective effects) besides promoting the regeneration of axons in sensory and motor neurons [23]. On the other hand, it is known that GDNF enhances the survival and outgrowth of both motor and sensory neurons [6] as well as stimulates axon sparing and regeneration [24]. NGF is neuroprotective and promotes neurite outgrowth of sensory and sympathetic neurons *in vitro* and *in vivo* [25]. Finally, VEGF is a factor known to promote angiogenesis but recent proofs indicate that this factor has a neurotrophic and neuroprotective effect besides stimulating axonal outgrowth [26]. In line with the previous studies herein performed, ASCs' gene expression seems to differ in each matrix (Figure 2), although no statistical differences were observed. However, it is of note that the outcome of the gene expression analysis should not be directly assumed to the presence of the protein itself. So, further studies comprising protein analysis should be performed to confirm these results.

Altogether, the data obtained on hydrogels' effect on ASC behavior indicate that a modulation of the secretome by the different matrices may be occurring and that ASCs adapt their behavior according to the surrounding environment and to the presence of biological cues. According to the results obtained, it was possible to observe that GG by itself is not able to promote DRG neurite outgrowth, while NVR and collagen promote a 15-fold increase of axonal growth in comparison to GG (Figure 4(a)). Interestingly, when ASCs are present in the 3D system (Figure 4(b)), NVR and collagen present an increase on DRG neurite outgrowth of only 0.2× and 0.5×, respectively, in comparison to the growth observed in these gels alone. On the other hand, a significant 10-fold increase was observed on GG hydrogels in the presence of ASCs. The normalization of the growth of axons on the hydrogels containing ASCs to the respective controls enables a clear interpretation of these results. The potentiation of neurite outgrowth by ASCs on GG can be explained by the higher level of cells' metabolic activity found within this hydrogel (Figure 1(f)). Consequently, cells might be able to secrete factors and molecules that help to compensate the initial difference observed between the three hydrogels. Altogether, these results point-out the GRGDS-GG matrix as the one to which ASCs adapt better and that potentiates their effects towards the promotion of axonal growth.

## 5. Conclusions

Spinal cord injury remains a physical, psychological, and economic problem, lacking regenerative therapies that translate into a complete and functional recovery of the patients. Numerous strategies have arisen for the treatment of SCI, but single therapy approaches were shown to be insufficient in successfully repairing the injured SC. For that reason, a combinatorial approach that acts in a synergistic arrangement can help overcome this problem, as it can enhance or avoid the limitations of the individual techniques. This work demonstrated that the use of hydrogels supports both the axonal growth and the ASC viability. The results from this study also revealed the importance of having biological cues, such as ECM molecules, to promote a more effective and pronounced effect on both the ASC and DRG cultures. Moreover, it appears that the ASCs vary their gene expression depending on the biological cues present on the hydrogels and that cells modulate their behaviour accordingly. A deeper understanding of the different interactions might give new insights for tissue engineering approaches and perhaps getting one step closer to a successful and functional recovery from SCI.

## Figures and Tables

**Table 1:** PCR primers used to detect gene expression in ASCs, upon seeding in the three hydrogels.

Gene	Primer sequence	Amplicon length
HMBS	Forward 5′-CCTGGCCCACAGCATACAT-3′ Reverse 5′-TCGGGGAAACCTCAACACC-3′	155 bp
GDNF	Forward 5′-AGCCGCTGCAGTACCTAAAA-3′ Reverse 5′-CCAACCCAGAGAATTCCAGA-3′	150 bp
VEGF	Forward 5′-TTTCTTGCGCTTTCGTTTTT-3′ Reverse 5′-AGGCCAGCACATAGGAGAGA-3′	133 bp
NGF	Forward 5′-GTCTGTGGCGGTGGTCTTAT-3′ Reverse 5′-CAACAGGACTCACAGGAGCA-3′	115 bp
BDNF	Forward 5′-AGAAGAGGAGGCTCCAAAGG-3′ Reverse 5′-TGGCTGACACTTTCGAACAC-3′	145 bp
